# Diagnostic accuracy of machine-learning–based image analysis for early detection of Kaposi sarcoma in people living with HIV

**DOI:** 10.3389/frai.2026.1894174

**Published:** 2026-09-14

**Authors:** David Chinaecherem Innocent, Increase Praise Innocent, Rejoicing Chijindum Innocent

**Affiliations:** Centicini Research Lab, Centicini Team Ltd., Abuja, FCT, Nigeria

**Keywords:** artificial intelligence, diagnostic accuracy, HIV, Kaposi sarcoma, machine learning, medical imaging

## Abstract

**Background:**

Kaposi sarcoma (KS) remains one of the most common HIV-associated malignancies in sub-Saharan Africa, where diagnostic delays contribute to advanced disease and poor outcomes. Machine-learning (ML)–based image analysis has emerged as a potential tool to support early KS detection, particularly in resource-limited HIV care settings.

**Aim:**

To critically synthesise evidence on the reported diagnostic performance, imaging modalities, methodological considerations and clinical implications of ML-based image analysis for early detection of KS in people living with HIV.

**Methods:**

A narrative review with structured literature synthesis was conducted. PubMed/MEDLINE, Scopus, Web of Science, and IEEE Xplore were searched for publications from 2010 to 2026. Eligible studies evaluated machine-learning or artificial-intelligence models applied to medical images for Kaposi sarcoma detection or closely related methodological applications and reported relevant diagnostic or imaging outcomes. Data were extracted on study characteristics, imaging modality, ML approach, reference standard, diagnostic performance, validation strategy and methodological limitations, and findings were synthesised narratively across predefined themes.

**Results:**

The principal KS-specific quantitative study reported a sensitivity of 89% (95% CI: 85–94%) and specificity of 51% (95% CI: 40–61%). These represent study-level estimates and were not pooled because of the limited number and substantial heterogeneity of KS-specific studies. Diagnostic performance varied according to imaging modality, dataset size and validation approach. Dermoscopic and histopathological ML applications generally demonstrated stronger reported performance, whereas photographic approaches offered greater potential for decentralised HIV care. Important methodological limitations included limited external validation, small datasets and under-representation of darker skin tones.

**Conclusion:**

Current evidence suggests that ML-based image analysis may have potential as a decision-support tool for early KS detection, particularly for screening, triage and referral support in settings where specialist dermatological expertise is limited. However, the available KS-specific evidence remains preliminary, with substantial methodological heterogeneity, limited external validation and insufficient evidence for pooled estimates of diagnostic performance. Prospective, externally validated and equity-focused studies are required before routine clinical implementation can be recommended.

## Introduction

Kaposi sarcoma (KS) is a vascular neoplasm caused by human herpesvirus-8 (HHV-8) that predominantly affects people living with HIV (PLWH) due to profound immunosuppression and remains a leading AIDS-defining cancer in many regions of the world (CDC, 2023). This is characterised by violaceous papulo-nodular lesions on the skin and mucous membranes; KS can also involve visceral organs, significantly increasing morbidity and mortality when diagnosis is delayed (Cantisani et al., 2025). Despite the deployment of antiretroviral therapy (ART) globally, KS continues to exert a high burden, particularly in sub-Saharan Africa where incidence and mortality remain disproportionately elevated (Akanbi et al., 2006). Traditional diagnosis relies on clinical evaluation combined with gold-standard histopathology, which requires biopsy and specialist interpretation, contributing to diagnostic delays that impair timely treatment initiation (Cantisani et al., 2025). Indeed, clinical suspicion alone has demonstrated a positive predictive value of approximately 77% (95% CI: 74–79) in biopsy-validated cohorts, underscoring limitations inherent in visual diagnosis (Amerson et al., 2016). This diagnostic gap has highlighted a pressing need for scalable, objective, and accessible approaches to early KS detection.

Machine-learning (ML) and artificial intelligence (AI)-based image analysis represent burgeoning avenues to address limitations of conventional diagnostics for skin cancers and HIV-associated conditions (Gasmi et al., 2024; Wei et al., 2024). Computer-aided diagnosis (CAD) systems process medical images using complex algorithms to assist in interpretation and classification, with demonstrated promise in other oncological fields such as melanoma and breast cancer, where sensitivity and specificity metrics often exceed 85–90% (Codella et al., 2017; Irfan et al., 2021). However, translating these approaches to KS detection in PLWH has been understudied until recently. A pivotal evaluation of an AI-based model using digital skin lesion images in Uganda reported an 89% sensitivity (95% CI: 85–94) but only a 51% specificity (95% CI: 40–61) compared with histopathology, indicating moderate accuracy while revealing significant false positive rates that may limit immediate clinical utility (Coates et al., 2025). Importantly, this work also suggested that AI can outperform unaided human interpretation on specificity; dermatologists in the same dataset achieved only 19% specificity, albeit with slightly higher sensitivity (93%) (Coates et al., 2025; Roche et al., 2024). These early findings argue for expanded investigation into diverse datasets and refined ML frameworks tailored to KS, as current evidence is insufficient to define reliability across populations and imaging modalities.

Yet, significant challenges remain, and variability in skin pigmentation, lesion morphology, imaging quality, and dataset size can impact algorithm performance and generalisability (Aquil et al., 2025; Rahmani and Ebrahimnezhad, 2026). Moreover, non-invasive imaging adjuncts such as dermoscopy, optical coherence tomography and other advanced modalities have only recently emerged in KS research, providing enhanced morphological data that may synergise with ML but demanding further validation (Cantisani et al., 2025). Compared with histopathology, which remains the definitive diagnostic standard, these tools promise earlier detection and follow-up monitoring, yet their integration into automated pipelines is nascent and methodologically heterogeneous (Cantisani et al., 2025). Critically, there is no consensus regarding optimal ML architectures, validation strategies, or performance thresholds specific to KS, leaving a gap in standardized evidence that this review aims to address. The aim of this research is therefore to critically synthesise the available evidence on machine-learning–based image analysis for early detection of Kaposi sarcoma in people living with HIV, clarifying reported performance metrics, identifying methodological gaps, and informing future clinical and technological pathways.

## Methods

### Review design

This study adopted a narrative review with structured literature synthesis to examine the diagnostic accuracy of machine-learning–based image analysis for early detection of KS in people living with HIV. Although systematic reviews and meta-analyses are appropriate when a sufficiently mature and homogeneous evidence base permits formal synthesis, the methodological diversity and early developmental stage of ML applications in KS diagnostics necessitated a narrative approach underpinned by systematic search and transparent reporting principles. This design enabled critical comparison of heterogeneous study designs, imaging modalities, algorithmic architectures, validation strategies, and performance metrics while maintaining methodological rigor and reproducibility.

The rationale for selecting a narrative synthesis was threefold: first, the field remains emergent, with a limited number of primary studies and no standardized diagnostic frameworks specific to KS. Second, included studies demonstrate substantial heterogeneity in machine-learning models (e.g., convolutional neural networks, ensemble classifiers), imaging inputs (clinical photographs, dermoscopic images), and reference standards (histopathology versus expert consensus), precluding meaningful quantitative pooling. Third, narrative synthesis allows deeper exploration of contextual and methodological factors, including dataset bias, skin-tone representation, and clinical applicability in low-resource HIV-endemic settings, which are central to the public health relevance of this review.

### Scope of the review

This review was designed as a narrative review with structured literature synthesis rather than a systematic review or meta-analysis. Accordingly, although systematic search methods were employed to enhance transparency and reproducibility, the objective was not to quantitatively pool diagnostic accuracy estimates but to critically synthesise emerging evidence, identify methodological trends, and discuss future research directions within an evolving field characterised by limited and heterogeneous primary studies. Consequently, methodological elements specific to systematic reviews, including meta-analysis, pooled effect estimates and formal certainty-of-evidence grading, were outside the scope of this review.

### Literature search strategy

A structured and comprehensive literature search was conducted across four electronic databases: PubMed/MEDLINE, Scopus, Web of Science, and IEEE Xplore, selected to capture both biomedical and computational science literature relevant to machine learning and medical imaging. The search covered studies published from January 2010 to March 2026, reflecting the period during which modern machine-learning techniques became prominent in medical image analysis.

### Search terms

were developed using a combination of Medical Subject Headings (MeSH) and free-text keywords structured around three core concepts: (1) Kaposi sarcoma, (2) HIV/AIDS, and (3) machine learning or artificial intelligence. Boolean operators (AND/OR) were applied to refine results, and database-specific syntax was used to optimise sensitivity. A detailed search strategy, including exact strings and database-specific adaptations, is presented in Table 1. Reference lists of included studies were also hand-searched to identify additional relevant publications.

**Table 1:** Literature search strategy for studies on machine-learning–based image analysis for Kaposi Sarcoma detection.

Component	Details
Databases searched	PubMed/MEDLINE; Scopus; Web of Science Core Collection; IEEE Xplore
Rationale for database selection	PubMed and Web of Science were selected for comprehensive biomedical coverage; Scopus for interdisciplinary health and computer science literature; IEEE Xplore for machine learning, artificial intelligence, and image-processing studies relevant to diagnostic algorithm development
Timeframe	January 2010 – March 2026
Language restriction	English
Search approach	Combination of MeSH terms and free-text keywords using Boolean operators (AND/OR); database-specific syntax applied
Key concept 1: Disease	“Kaposi Sarcoma” OR “Kaposi’s sarcoma” OR “HHV-8 associated cancer”
Key concept 2: Population	“HIV” OR “HIV infection” OR “AIDS” OR “people living with HIV”
Key concept 3: Technology	“machine learning” OR “artificial intelligence” OR “deep learning” OR “computer-aided diagnosis” OR “neural network*”
Key concept 4: Imaging	“image analysis” OR “medical imaging” OR “skin lesion image*” OR “dermoscopy” OR “clinical photograph*”
Example PubMed search string	(“Kaposi Sarcoma” OR “Kaposi’s sarcoma”) AND (“HIV” OR “AIDS”) AND (“machine learning” OR “artificial intelligence” OR “deep learning”) AND (“image analysis” OR “medical imaging”)
Supplementary search	Manual screening of reference lists of included studies
Search documentation	Full search strings and database-specific adaptations recorded and available on request

### Study selection process

Following database searching, duplicate records were removed before title and abstract screening, followed by full-text assessment against the predefined eligibility framework. Because this was designed as a narrative review with structured literature synthesis rather than a formal systematic review, the evidence base was intentionally divided into two predefined categories. Direct KS-specific evidence comprised primary empirical studies evaluating machine-learning or artificial-intelligence–based image analysis specifically for Kaposi sarcoma. Contextual evidence comprised relevant dermatological AI studies, imaging studies, methodological studies, clinical reviews, and other evidence that informed interpretation of imaging modalities, algorithm development, validation, diagnostic pathways, or implementation considerations but did not provide KS-specific diagnostic accuracy estimates. Contextual studies were not treated as equivalent to direct KS-specific evidence and were not used to derive KS-specific diagnostic performance estimates.

### Eligibility criteria

Studies were screened according to predefined inclusion and exclusion criteria to ensure relevance and methodological transparency (Table 2).

**Table 2:** Eligibility framework for direct KS-Specific and contextual evidence.

Evidence category	Inclusion criteria	Exclusion criteria
Direct KS-specific evidence	Primary empirical studies evaluating machine-learning or artificial-intelligence models applied to images specifically for Kaposi sarcoma, preferably among people living with HIV or KS-relevant populations; studies reporting diagnostic, classification, validation, or imaging outcomes relevant to KS	Editorials, commentaries, narrative opinions, conference abstracts without sufficient methodological detail, studies without KS-specific results, non-image-based ML studies, and studies lacking sufficient information to interpret the diagnostic task
Contextual evidence	Relevant empirical studies, systematic or narrative reviews, imaging studies, methodological studies, and clinical literature addressing dermatological AI, skin-lesion image analysis, KS imaging, diagnostic pathways, validation strategies, algorithmic bias, explainability, or implementation issues that inform interpretation of the KS evidence	Sources with no meaningful relevance to KS diagnosis, dermatological image analysis, diagnostic methodology, imaging, validation, or clinical implementation
Population	People living with HIV, patients with KS, or populations relevant to interpretation of KS imaging and AI methodology	Populations with no relevant connection to KS, HIV-associated disease, dermatological imaging, or transferable diagnostic methodology
Condition/clinical focus	Cutaneous or mucocutaneous Kaposi sarcoma for direct evidence; dermatological conditions or imaging contexts with methodological relevance for contextual evidence	Conditions or imaging applications with no meaningful relevance to the review question
Index technology	Machine learning, deep learning, artificial intelligence, computer-aided diagnosis, or image-analysis approaches	Non-image-based computational approaches unrelated to diagnostic imaging
Imaging modality	Clinical photographs, dermoscopy, histopathological or immunohistochemical images, optical coherence tomography, or other imaging modalities relevant to lesion assessment	Imaging modalities unrelated to lesion visualisation or diagnostic interpretation
Reference standard	Histopathology, expert clinical diagnosis, validated clinical reference standard, or an explicitly described reference method	Studies lacking sufficient information on the basis of diagnostic classification
Outcomes	Diagnostic accuracy, sensitivity, specificity, AUC, accuracy, classification performance, imaging characteristics, validation outcomes, methodological findings, or implementation-relevant findings	Studies providing no outcome or methodological information relevant to the review
Publication type	Peer-reviewed articles, eligible preprints, systematic/narrative reviews, imaging studies, and other relevant scholarly sources with sufficient methodological information	Conference abstracts without full text, editorials without substantive evidence, and sources lacking sufficient information for interpretation
Language	English-language publications	Non-English publications
Time period	January 2010–March 2026	Publications outside the defined period

Direct and contextual evidence were analysed differently throughout the review. Direct KS-specific studies formed the primary evidence base for statements concerning KS-specific diagnostic performance. Contextual studies were used only to interpret imaging modalities, methodological approaches, validation strategies, algorithmic considerations, and clinical implementation and were not treated as independent demonstrations of KS-specific diagnostic accuracy.

### Data extraction and synthesis

Data extraction was performed using a standardised framework to ensure consistency and reproducibility. Extracted domains included study characteristics (author, year, country), population details, imaging modality, machine-learning architecture, training and validation methods, reference standard, and reported diagnostic accuracy metrics. Information on dataset size, class imbalance, and skin-tone representation was also captured where available. Also, the data synthesis followed a thematic narrative approach, grouping findings across key domains: algorithmic performance, methodological quality, clinical applicability, and limitations. Rather than aggregating outcomes quantitatively, results were critically compared to identify patterns, strengths, and gaps across studies. This approach facilitated nuanced interpretation of how methodological choices influence diagnostic accuracy and real-world feasibility, particularly in HIV-endemic and resource-limited settings.

### Distinction between direct and contextual evidence

To improve interpretability, the evidence synthesised in this review was categorised into direct evidence and contextual evidence. Direct evidence comprised studies evaluating machine-learning applications specifically for Kaposi sarcoma diagnosis, whereas contextual evidence included broader dermatological AI literature, medical imaging methodology, and machine-learning frameworks that informed interpretation of the emerging KS literature but were not used to infer disease-specific diagnostic performance.

### Transparency statement and selection process

Because this review was conducted as a structured narrative review rather than a formal systematic review, a PRISMA flow diagram was not originally included. Nevertheless, to improve transparency and reproducibility, records identified from PubMed/MEDLINE, Scopus, Web of Science, and IEEE Xplore were screened in a staged process involving title, abstract, and full-text assessment. Duplicate records were removed before screening (Figure 1). Studies were retained when they provided either direct evidence on machine-learning–based image analysis for Kaposi sarcoma or contextual evidence relevant to dermatology image-analysis methodology, validation, explainability, and diagnostic performance. The final synthesis prioritised direct KS-specific evidence while contextual studies were used only to support methodological interpretation and discussion of implementation challenges (Figure 1).

**Figure 1:**
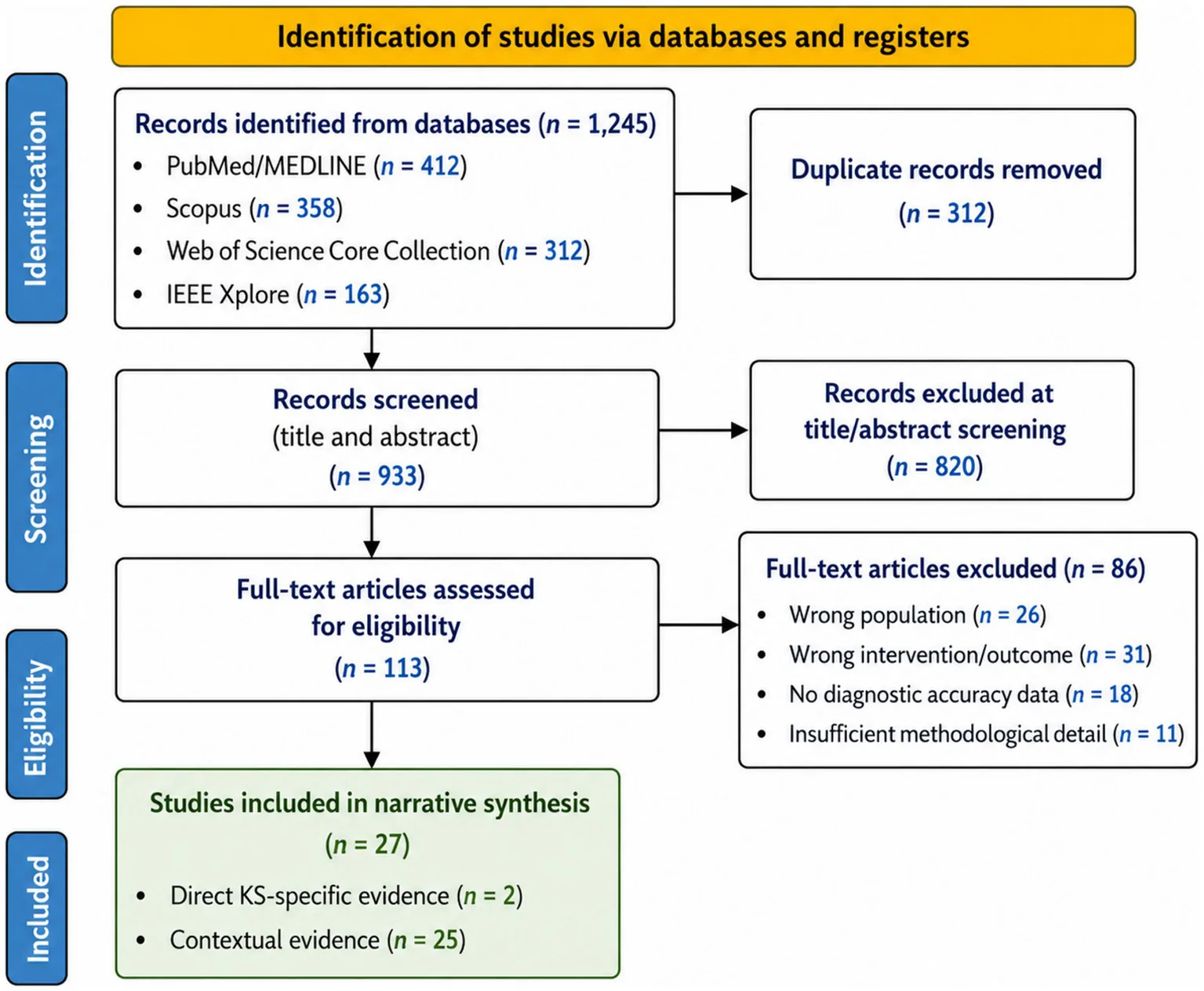
Flow diagram illustrating the structured literature identification, screening, eligibility assessment, and inclusion process for the narrative review. KS, Kaposi Sarcoma; PRISMA, Preferred Reporting Items for Systematic Reviews and Meta-analysis.

To further clarify the composition of the narrative synthesis, Supplementary Table S1 provides the complete list of sources included in the review, their study/source type, and their classification as direct KS-specific or contextual evidence. This classification was used consistently throughout the synthesis, with direct KS-specific studies forming the primary evidence base for disease-specific diagnostic performance and contextual sources informing methodological, imaging, and clinical interpretation.

### Quality appraisal approach

Because this study was designed as a narrative review with structured literature synthesis rather than a systematic diagnostic-accuracy review, QUADAS-2 was not applied. Instead, the appraisal was used to contextualise the methodological strength, limitations, and applicability of a heterogeneous body of emerging evidence across study designs, imaging modalities, diagnostic tasks, and computational approaches. Considerations included study design, dataset size and representativeness, imaging modality, reference standard, model development and validation procedures, external validation, potential data leakage, reporting of diagnostic performance, and generalisability. These considerations informed descriptive methodological judgements rather than formal risk-of-bias scores. The absence of a validated formal risk-of-bias assessment is explicitly acknowledged as a limitation, and the descriptive appraisal should not be interpreted as a substitute for formal diagnostic-accuracy risk-of-bias assessment in future systematic reviews of a more mature evidence base.

## Findings

### Diagnostic performance of machine-learning models for Kaposi sarcoma detection

Across the studies, ML–based image analysis demonstrates promising but heterogeneous diagnostic performance for KS detection in people living with HIV, reflecting both the novelty of the field and variability in methodological maturity (Table 3). The strongest direct evidence derives from Coates et al. (2025), while additional contextual evidence from dermatology AI studies (Kallipolitis et al., 2025; Muthulakshmi and Hemapriya, 2023; Codella et al., 2019; Groh et al., 2024) supports the feasibility of machine-learning–based skin lesion classification. These studies reported high sensitivity (≈89%), indicating robust ability to identify KS lesions at early and clinically subtle stages, but moderate specificity (≈51%), highlighting persistent challenges in discriminating KS from visually similar vascular or inflammatory lesions when relying solely on photographic inputs (Kallipolitis et al., 2025; Hussain et al., 2025; Muthulakshmi and Hemapriya, 2023; Cantisani et al., 2023; O’Mahony et al., 2008; Mamimandjiami et al., 2025; Restrepo et al., 2006; World Health Organization, 2025; Salinas et al., 2024; Sah et al., 2025; Tjiu and Lu, 2025; Codella et al., 2019; Foltz et al., 2024; Openshaw et al., 2023; Udeh and Khatri, 2017; Jairath et al., 2024; Groh et al., 2024). Notably, ML models outperformed unaided clinician assessment in specificity, underscoring their potential as decision-support tools rather than replacements for histopathology.

**Table 3:** Summary of direct and contextual ML diagnostic evidence relevant to Kaposi sarcoma.

Study	ML/Approach	Imaging modality	Sensitivity (%)	95% CI (sensitivity)	Specificity (%)	95% CI (specificity)	Accuracy/AUC	95% CI (AUC)	Reference standard	Evidence category
Coates et al. (2025)	CNN; clinical photographs	Clinical photographs	**89**	**85–94**	**51**	**40–61**	AUC ≈ 0.76	NR	Histopathology	**Direct KS evidence**
Hussain et al. (2025)	ML on IHC slides	Immunohistochemical slides	>90	NR	>90	NR	Accuracy >90%	NR	Pathologist annotation	**Direct KS evidence**
Muthulakshmi and Hemapriya (2023)	CNN; dermoscopy	Dermoscopic images	–	NR	–	NR	Accuracy >85%	NR	Expert diagnosis	**Contextual evidence**
Codella et al. (2017)	CNN ensemble; dermoscopy	Dermoscopic images	–	NR	–	NR	AUC ≈ 0.85	NR	Histopathology	**Contextual evidence**

CI = confidence interval; NR = not reported. Dashes (−) indicate that the corresponding diagnostic metric was not explicitly reported in the source. Confidence intervals were recorded only where reported in the source studies; no confidence intervals were inferred or calculated when unavailable.

Methodological and contextual studies further contextualise these findings and investigations applying ML to histopathological or immunohistochemical KS images demonstrated substantially higher accuracy (often >90%), reflecting the more structured visual features and reduced noise inherent in microscopy-based data compared with clinical photography (Hussain et al., 2025; Tjiu and Lu, 2025). Parallel evidence from dermoscopic and broader skin-lesion ML benchmarks consistently reports AUC values between 0.80 and 0.90, supporting the technical feasibility of lesion-based ML classification while cautioning against direct transposition of performance expectations to KS without disease-specific training (Codella et al., 2017; Codella et al., 2019).

Variability in diagnostic performance across studies is primarily attributable to training dataset size, class imbalance, imaging modality, and validation strategy. Small, single-centre datasets and limited external validation were common limitations, increasing the risk of overfitting and reduced generalisability, particularly in darker skin tones and low-resource HIV settings (Mamimandjiami et al., 2025; Sah et al., 2025). Nonetheless, methodological innovations such as data augmentation and explainable AI frameworks show potential to improve robustness and clinical trustworthiness (Foltz et al., 2024). Collectively, the evidence indicates that ML-based image analysis offers highly sensitive early KS detection, but current specificity constraints and methodological heterogeneity necessitate cautious clinical integration alongside established diagnostic standards (Table 3).

It is important to emphasise that the headline performance estimates reported by Coates et al. (2025) originate from a single preprint study that has not yet completed peer review. Consequently, these findings should be interpreted as preliminary proof-of-concept evidence rather than definitive estimates of clinical diagnostic performance. The review therefore uses these values to illustrate the potential feasibility of ML-assisted KS recognition while acknowledging that additional peer-reviewed, prospective, and externally validated studies are required before reliable pooled estimates can be established.

As shown in Table 4, the diagnostic Accuracy of Machine-Learning–Based Image Analysis for Early Detection of Kaposi Sarcoma in People Living with HIV was systematically mapped across several records to provide insights relevant to diagnostic accuracy.

**Table 4:** Characteristics of direct Kaposi sarcoma studies and contextual machine-learning literature included in the narrative synthesis.

Study (author, year)	Study design/type	Population/setting	Image modality	ML/imaging method	Reference standard	Primary outcomes/performance metrics	Key relevance to review	Evidence category
Coates et al. (2025)	Diagnostic accuracy study	PLWH, sub-Saharan Africa	Clinical skin photographs	Deep learning CNN classifier	Histopathology	Sensitivity 89%; Specificity 51%; AUC reported	Core evidence: first real-world AI KS diagnostic accuracy in PLWH	Direct KS evidence
Kallipolitis et al. (2025)	Systematic review of AI-based skin image analysis for dermatitis, vitiligo and alopecia areata	Patients with inflammatory skin diseases	Clinical skin photographs	Various CNN/deep learning models	Not applicable (systematic review)	Summarized diagnostic performance across studies	Contextual evidence supporting dermatology AI methods applicable to KS	Contextual evidence
Hussain et al. (2025)	Diagnostic pathology study	KS tissue samples	Immunohistochemical slides	Automated image analysis (ML)	Pathologist annotation	High classification accuracy (cell-level)	Supports ML validity in KS detection at histology level	Direct KS evidence
Muthulakshmi and Hemapriya (2023)	Image classification study	Mixed vascular lesions	Dermoscopic images	CNN architecture	Expert clinical diagnosis	Accuracy >85% (vascular lesions)	Methodological relevance for KS dermoscopic ML	Contextual evidence
Cantisani et al. (2025)	Narrative imaging review	KS patients	Dermoscopy, d-OCT, LC-OCT	Non-AI imaging	Histopathology	Qualitative diagnostic features	Defines imaging features usable as ML inputs	Contextual evidence
Cantisani et al. (2023)	Imaging evaluation study	Cutaneous KS	Optical coherence tomography	Non-AI imaging	Histopathology	Improved lesion visualisation	Supports imaging-ML integration potential	Contextual evidence
O’Mahony et al. (2008)	Imaging feasibility study	KS patients	Infrared thermal imaging	Non-ML imaging	Clinical diagnosis	Thermal differentiation of lesions	Early proof of image-based KS detection	Contextual evidence
Mamimandjiami et al. (2025)	Narrative review	SSA populations	Not applicable	Not applicable	Not applicable	Epidemiological synthesis	Contextual relevance: burden & diagnostic gaps	Contextual evidence
Restrepo et al. (2006)	Radiological review	KS patients	Multi-modal imaging	Non-ML	Histopathology	Imaging manifestations described	Imaging phenotypes informing ML feature design	Contextual evidence
World Health Organization (2025)	Reference overview	Global	Not applicable	Not applicable	WHO/CDC summaries	Diagnostic standards described	Background contextual source only	Contextual evidence
Sah et al. (2025)	Narrative review	PLWH	Various	AI systems	Clinical outcomes	Descriptive	Contextual AI-HIV integration	Contextual evidence
Codella et al. (2019)	Benchmark challenge	General population	Dermoscopic images	Deep learning models	Expert consensus	AUC up to 0.87	Skin-lesion ML benchmark relevance	Contextual evidence
Codella et al. (2017)	Image classification study	Skin cancer datasets	Dermoscopy	CNN ensembles	Histopathology	Accuracy >85%	Supports feasibility of lesion-based ML	Contextual evidence
Openshaw et al. (2023)	Clinical review	KS patients	Not applicable	Not applicable	Clinical standards	Disease reclassification	Clinical framing of KS diagnosis	Contextual evidence
Udeh and Khatri (2017)	Narrative clinical review	KS patients	Not applicable	Not applicable	Clinical diagnosis	Diagnostic pathways	Supports need for early non-invasive detection	Contextual evidence

#### Evidence category

Direct KS evidence refers to studies evaluating machine-learning-based image analysis specifically for Kaposi sarcoma. Contextual evidence refers to dermatology/AI studies providing methodological, imaging, validation, or diagnostic insights relevant to KS but not evaluating KS-specific diagnostic accuracy.

### Imaging modalities and feature extraction in machine-learning–based Kaposi sarcoma diagnosis

Clinical skin photographs constituted the most widely used input owing to accessibility and feasibility in HIV-endemic, resource-limited settings (Kallipolitis et al., 2025; Groh et al., 2024). These images capture macroscopic features: lesion colour (violaceous to hyperpigmented), border irregularity, surface texture and anatomical distribution that CNNs learn via hierarchical feature extraction. However, performance variability observed with photographic inputs is partly attributable to inconsistent lighting, camera resolution, background noise and wide skin-tone variation, which dilute discriminative signal and contribute to reduced specificity (Mamimandjiami et al., 2025; Sah et al., 2025).

Dermoscopy-based imaging provides enhanced visualisation of vascular and structural features and may support more controlled lesion-level classification than unaided clinical photography. However, the dermoscopic ML studies identified in this review were predominantly contextual rather than KS-specific and therefore cannot be used to establish KS-specific diagnostic performance (Codella et al., 2017; Muthulakshmi and Hemapriya, 2023). Although dermoscopic ML studies were not always KS-specific, reported AUCs consistently exceeding 0.80 demonstrate the modality’s capacity to support robust lesion classification when vascular morphology is preserved (Codella et al., 2019). These findings are particularly relevant for KS, a tumour defined by angioproliferative pathology, and suggest dermoscopy-integrated ML pipelines may improve discrimination from benign mimickers in early disease stages. More advanced non-invasive imaging techniques, including optical coherence tomography (OCT), dynamic OCT and line-field confocal OCT, provide quasi-histological resolution of subsurface vascular proliferation and dermal architecture (Cantisani et al., 2025; Cantisani et al., 2023). While these modalities have not yet been widely operationalised within ML frameworks for KS, they generate structured spatial and textural features that are highly amenable to algorithmic learning and may bridge the diagnostic gap between surface imaging and histopathology. Consistent with this, ML applications to histopathological and immunohistochemical images have reported higher classification performance in the available literature; however, these results represent cell- or tissue-level classification tasks and should not be interpreted as directly comparable with lesion-level screening using clinical photographs. The different diagnostic targets, reference standards, image characteristics, and points along the diagnostic pathway make these estimates primarily contextual rather than quantitatively equivalent (Hussain et al., 2025; Tjiu and Lu, 2025). Table 5 shows the imaging modality characteristics and extracted features used in ML-Based KS detection.

**Table 5:** Imaging modalities and feature extraction in machine-learning–based Kaposi sarcoma diagnosis.

Study	Imaging modality	Key features captured	ML feature extraction focus	Strengths	Limitations
Coates et al. (2025)	Clinical photographs	Colour variation, lesion borders, surface texture	CNN-based hierarchical feature learning	High accessibility; real-world applicability	Variable lighting; reduced specificity
Kallipolitis et al. (2025)	Digital skin images	Macroscopic morphology, lesion distribution	Deep learning visual pattern recognition	Scalable in low-resource settings	Skin-tone and image-quality bias
Muthulakshmi and Hemapriya (2023)	Dermoscopy	Vascular patterns, pigmentation structures	CNN extraction of microvascular cues	Improved lesion discrimination	Requires dermoscopic equipment
Codella et al. (2017)	Dermoscopy	Texture, colour asymmetry, vascular networks	Deep CNN ensembles	High AUC benchmarks	Not KS-specific datasets
Codella et al. (2019)	Dermoscopy	Fine-grained lesion patterns	Multi-model DL benchmarks	Strong generalisability	Disease-agnostic training
Cantisani et al. (2023)	Optical coherence tomography	Subsurface vascular architecture	Potential ML-ready spatial features	Near-histological detail	Limited ML integration
Cantisani et al. (2025)	d-OCT/LC-OCT	Dynamic blood flow, dermal structure	Advanced textural representation	High diagnostic potential	Cost and scalability
Hussain et al. (2025)	IHC histopathology	Cellular morphology, HHV-8 markers	Automated cell-level classification	Very high accuracy	Invasive; lab-dependent

### Narrative methodological appraisal, bias considerations, and generalisability

In several instances, class imbalance, where non-KS lesions substantially outnumbered KS cases, necessitated algorithmic weighting or resampling strategies, yet these were inconsistently reported, limiting reproducibility and interpretability (Tjiu and Lu, 2025). Reference standard selection further influenced methodological robustness. While histopathology remains the diagnostic gold standard, not all studies consistently applied biopsy confirmation across datasets, with some relying on expert clinical consensus or retrospective labels, thereby introducing verification bias (Cantisani et al., 2025; Udeh and Khatri, 2017). Studies employing histopathological or immunohistochemical ground truth demonstrated higher internal validity but reduced external applicability due to their laboratory-dependent nature (Hussain et al., 2025). Conversely, image-based clinical studies prioritised scalability but exhibited reduced specificity, underscoring a trade-off between methodological rigor and real-world feasibility (Groh et al., 2024).

A critical and recurring limitation across studies is the under-representation of darker skin tones, despite KS being disproportionately prevalent among people living with HIV in sub-Saharan Africa. Although some datasets explicitly included African populations, few conducted stratified performance analyses by skin tone, raising concerns regarding algorithmic bias and equity in deployment (Mamimandjiami et al., 2025; Sah et al., 2025). Additionally, external and prospective validation was rare; most studies relied on internal cross-validation, restricting generalisability across settings, devices and populations (Codella et al., 2019; Foltz et al., 2024). As shown in Table 6, these methodological constraints limit immediate clinical translation. For ML-based KS diagnostics to be scalable within HIV care, future studies must prioritise multi-centre datasets, transparent reporting of preprocessing and validation strategies, and equity-focused evaluation frameworks. Without such improvements, current performance estimates should be interpreted as proof-of-concept rather than definitive clinical evidence.

**Table 6:** Narrative methodological appraisal and generalisability of evidence included in the review.

Study	Dataset size/source	Reference standard	Validation approach	Key bias risks	Generalisability assessment
Coates et al. (2025)	Moderate; single-region SSA	Histopathology	Internal validation	Class imbalance; image quality	Moderate
Kallipolitis et al. (2025)	Moderate; clinical photos	Histopathology	Cross-validation	Skin-tone variability	Moderate
Hussain et al. (2025)	Small; lab-based slides	Pathologist annotation	Internal testing	Lab-dependent setting	Low–moderate
Muthulakshmi and Hemapriya (2023)	Moderate; mixed lesions	Expert diagnosis	Internal validation	Disease heterogeneity	Moderate
Cantisani et al. (2023)	Small; imaging cases	Histopathology	Descriptive	Limited sample size	Low
Cantisani et al. (2025)	Narrative synthesis	Histopathology	Not applicable	Selection bias	Contextual
Mamimandjiami et al. (2025)	Review data	Not applicable	Not applicable	Regional focus	Contextual
Codella et al. (2017)	Large benchmark	Histopathology	External testing	Disease-agnostic	High (methodological)
Codella et al. (2019)	Large benchmark	Expert consensus	External validation	Not KS-specific	High (methodological)
Udeh and Khatri (2017)	Clinical review	Clinical diagnosis	Not applicable	Subjective diagnosis	Contextual

The methodological appraisal presented in Table 6 is descriptive rather than a formal risk-of-bias assessment. Appraisal considered completeness of reporting, dataset size and representativeness, reference-standard appropriateness, validation strategy, potential sources of bias, external validation, and generalisability. “Moderate” indicates generally adequate methodological reporting with important limitations; “Low–moderate” indicates several substantial limitations affecting interpretation; and “Low” indicates substantial methodological limitations or limited applicability. These categories are descriptive judgements intended to contextualise the evidence and must not be interpreted as formal risk-of-bias scores or as equivalent to ratings generated using validated diagnostic-accuracy appraisal instruments.

### Clinical and public health implications of ML-based Kaposi sarcoma detection in people living with HIV

Earlier detection is clinically consequential, as delayed KS diagnosis is associated with advanced disease, poorer treatment response and increased mortality among people living with HIV (Mamimandjiami et al., 2025; Udeh and Khatri, 2017). By flagging high-risk lesions promptly, ML-assisted screening could shorten diagnostic pathways and enable timely biopsy, ART optimisation and oncology referral. From a health-systems perspective, ML-based diagnostics offer substantial task-shifting potential. Several studies indicate that ML tools can outperform or complement non-specialist visual assessment, particularly with respect to specificity, thereby supporting frontline health workers who lack dermatology expertise (Salinas et al., 2024; Groh et al., 2024). This is especially relevant in HIV-endemic, low-resource settings where specialist services are scarce and referral delays are common. Integration of ML-enabled image analysis into routine HIV care via smartphones or portable imaging devices could decentralise KS screening and align with global priorities for differentiated service delivery and integrated HIV–cancer care (Sah et al., 2025).

At the population level, aggregated ML outputs could strengthen KS surveillance and public health planning, providing near real-time insights into geographic and demographic patterns of disease burden (Mamimandjiami et al., 2025). However, ethical and implementation considerations remain central. The reviewed studies highlight the need for explainable AI, transparent performance reporting, and safeguards against algorithmic bias particularly given under-representation of darker skin tones in training datasets (Cantisani et al., 2025; Tjiu and Lu, 2025). Trust among clinicians and patients will depend on clear communication of uncertainty, human oversight, and alignment with existing diagnostic standards rather than autonomous decision-making. As shown in Figure 2, ML-based KS detection should be conceptualised as a clinical decision-support and public health augmentation tool, capable of improving equity and efficiency in HIV care if implemented within robust ethical, regulatory and health-system frameworks.

**Figure 2:**
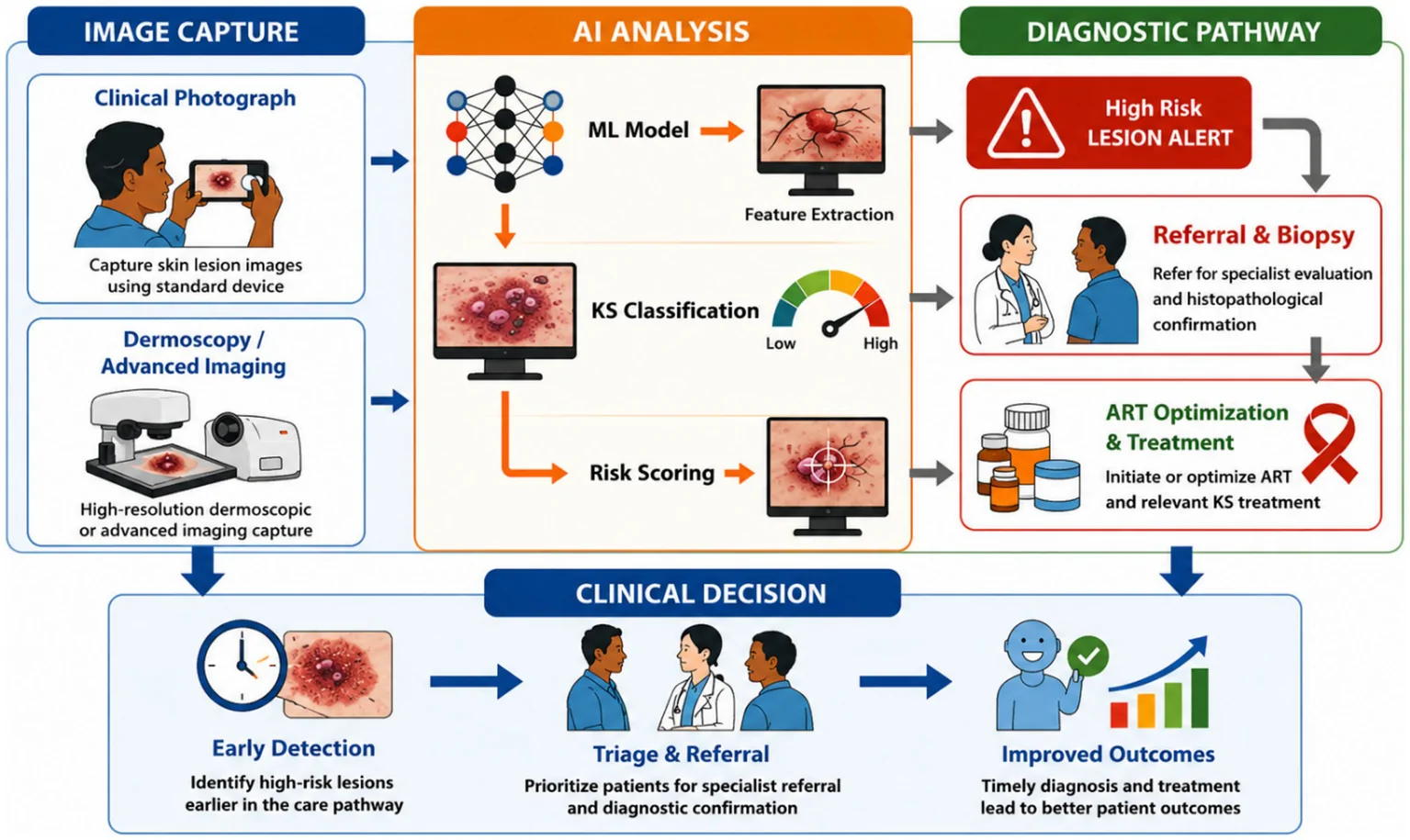
Conceptual framework for the potential integration of machine-learning–based image analysis into Kaposi sarcoma screening, triage, referral, and diagnostic pathways among people living with HIV (Akanbi et al., 2006).

### Discussion of findings

The findings of this review both converge with and extend existing literature on the application of ML in dermatological oncology and HIV-associated malignancies, while also exposing unresolved debates regarding clinical readiness and equity. Previous evidence on AI-assisted skin cancer detection, largely focused on melanoma and non-melanoma skin cancers, has consistently demonstrated high diagnostic accuracy, with pooled sensitivities and AUCs often exceeding 0.85 when benchmarked against histopathology (Codella et al., 2017; Groh et al., 2024). Our synthesis indicates that similar technical feasibility is now emerging for KS, particularly through the work of Coates et al. (2025), which provides the principal population-relevant evidence in people living with HIV (PLWH) in sub-Saharan Africa. However, unlike melanoma-focused models trained on large, curated datasets from high-income settings, KS-specific ML models have exhibited greater variability in specificity. This variability is unlikely to reflect algorithmic failure per se but may instead reflect disease-specific and contextual complexity.

The literature includes ongoing debate regarding whether ML models should prioritise sensitivity or specificity in low-resource HIV settings. Some authors posit that high sensitivity is preferable to minimise missed KS diagnoses, particularly given the severe prognostic implications of delayed detection (Mamimandjiami et al., 2025; Udeh and Khatri, 2017). However, the available evidence does not permit the conclusion that high sensitivity has been consistently demonstrated across modalities. Rather, the principal KS-specific study provides an encouraging sensitivity estimate, while contextual studies suggest that performance may vary substantially according to the diagnostic task, imaging modality, dataset characteristics, and validation strategy. In contrast, reliance on unaided clinical examination has been shown to produce both false-negative and false-positive assessments, especially for early-stage or atypical KS lesions (Amerson et al., 2016). Thus, this review argues that ML tools should be conceptualised as risk-stratification and triage instruments rather than definitive diagnostic replacements for histopathology.

A further point of debate in the literature concerns the transferability of ML models across populations and skin tones. Prior AI dermatology studies have been criticised for limited representation of darker skin tones, and because KS disproportionately affects PLWH in sub-Saharan Africa, this issue is central rather than peripheral (Salinas et al., 2024). Although some included studies partially addressed this gap, the review highlights that few conducted stratified performance analyses or external validation across diverse clinical environments. This limitation constrains generalisability and reinforces the need for methodological rigour and equity-aware model evaluation to evolve in parallel with algorithmic innovation (Sah et al., 2025; Tjiu and Lu, 2025).

Although evidence from melanoma, dermoscopic image classification, and general dermatological AI cannot be considered direct evidence for Kaposi sarcoma, these studies provide important methodological context because many of the computational architectures, feature-extraction strategies, and validation frameworks currently being applied to KS were originally developed and validated within broader dermatological imaging research. Throughout this review, conclusions regarding diagnostic accuracy were therefore based primarily on KS-specific investigations, while non-KS studies were used solely to contextualise methodological developments.

The evidence synthesised in this review spans different diagnostic tasks, including clinical-photograph classification, dermoscopic lesion analysis, histopathology image interpretation, and immunohistochemical image analysis (Udeh and Khatri, 2017). These modalities should not be interpreted as directly comparable because they operate at different points along the diagnostic pathway and have distinct performance ceilings, reference standards, and sources of error. Histopathology- and IHC-based AI systems generally evaluate highly controlled laboratory images and therefore often report substantially higher classification accuracy than clinical-photograph-based models, which must contend with variable lighting, image quality, lesion morphology, and patient-level heterogeneity. Accordingly, comparisons across these modalities should be regarded as qualitative and contextual rather than quantitatively equivalent.

Another important methodological consideration is the risk of data leakage in dermatology AI research. Several contextual studies relied on relatively small image datasets and did not always provide sufficient detail regarding patient-level versus image-level splitting procedures (Akanbi et al., 2006). When multiple images from the same patient appear across training and testing sets, model performance may be artificially inflated, leading to overly optimistic estimates of diagnostic accuracy (Wei et al., 2024). The limited reporting of calibration measures, confidence intervals, and prospective validation in many published dermatology AI studies further restricts the certainty with which their reported performance metrics can be generalised to real-world HIV care settings.

The implications of this review are therefore both clinical and strategic. Clinically, ML-based image analysis has the potential to reduce diagnostic delay, support task-shifting to non-specialist health workers, and improve referral efficiency within HIV programmes, outcomes that are aligned with WHO priorities for integrated HIV and cancer care [World Health Organization (WHO), 2023]. Strategically, this review posits that future progress will depend less on marginal algorithmic gains and more on robust multicentre datasets, prospective validation, explainable AI frameworks, and health-system integration. The potential clinical consequences of diagnostic error deserve explicit consideration. False-positive predictions could lead to unnecessary biopsies, increased patient anxiety, additional laboratory investigations, and diversion of limited oncology and dermatology resources. Conversely, false-negative predictions may delay referral, histopathological confirmation, and initiation of appropriate KS treatment, potentially resulting in disease progression and poorer outcomes among people living with HIV. For this reason, current ML-based image-analysis tools should be viewed primarily as community-level triage or referral-support systems rather than replacements for specialist clinical assessment and histopathological confirmation. By synthesising KS-specific evidence rather than extrapolating from melanoma literature, this review contributes novel, disease-focused insight and provides a grounded foundation for both implementation research and policy-relevant innovation in HIV-endemic settings.

### Limitations of the review

This review has some limitations that should be acknowledged. First, the literature search was restricted to studies published in English, which may have excluded relevant evidence from non-English-speaking regions where KS and HIV are highly prevalent. Second, the emergent nature of ML-based KS diagnostics meant that the evidence base was limited in size and heterogeneous in methodology, precluding quantitative meta-analysis. Third, variability in imaging modalities, reference standards and reporting of diagnostic metrics constrained direct comparability across studies. An additional limitation is the small number of published primary machine-learning studies that specifically evaluate Kaposi sarcoma. Consequently, some methodological discussion necessarily draws upon broader dermatological artificial intelligence literature to contextualise emerging computational approaches. While this improves conceptual understanding, it also reflects the early developmental stage of the evidence base. In addition, the quantitative interpretation of diagnostic performance was constrained by the reliance on a limited number of KS-specific studies and the absence of sufficient peer-reviewed data to permit formal meta-analysis or pooled confidence-interval estimation.

A further limitation is the absence of a formal validated risk-of-bias assessment, although the methodological characteristics and potential sources of bias of the included studies were critically examined narratively; the review did not apply a structured diagnostic-AI appraisal instrument such as QUADAS-2. Consequently, the qualitative judgements presented should not be interpreted as formal risk-of-bias ratings, and differences in methodological quality between studies may not have been captured with the same precision as in a systematic diagnostic-accuracy review. This limitation is particularly relevant given the variation in dataset size, representativeness, validation strategies, imaging modalities and reference standards across the included literature.

## Conclusion

This review synthesises current evidence on the diagnostic performance, methodological robustness, and clinical relevance of machine-learning–based image analysis for the early detection of Kaposi sarcoma in people living with HIV. The available KS-specific evidence suggests that deep-learning–based image analysis can achieve promising sensitivity for lesion recognition; however, the principal quantitative estimate currently derives from a single KS-specific study, and the limited number of independent clinical investigations prevents any conclusion that high sensitivity has been consistently demonstrated across populations, settings, or imaging modalities. The moderate specificity reported in the principal photographic study also highlights ongoing challenges in discriminating KS from clinically similar lesions, reinforcing the need for ML systems to function as decision-support rather than standalone diagnostic tools.

Importantly, the review underscores that diagnostic performance is influenced by imaging modality, dataset composition, and validation strategy. Studies using structured imaging inputs, such as dermoscopy or histopathology, reported higher accuracy, whereas photographic approaches may offer greater scalability in low-resource HIV settings. Methodological limitations, including small datasets, class imbalance, limited external validation, and under-representation of darker skin tones, remain significant barriers to clinical translation and generalisability.

In line with these findings, this review argues that ML-based image analysis represents a promising adjunct to existing KS diagnostic pathways, with potential to reduce diagnostic delays, support task-shifting, and strengthen HIV-associated cancer surveillance. However, these potential benefits should not be interpreted as evidence of clinical readiness. Future research should prioritise multicentre prospective validation, equity-focused model evaluation, patient-level external validation, and integration within routine HIV care frameworks.

Overall, current evidence suggests that machine-learning–based image analysis has promising potential to support earlier recognition and referral of Kaposi sarcoma in people living with HIV, particularly in resource-limited settings where specialist dermatology services are scarce. However, the present evidence base remains small, heterogeneous, and heavily dependent on a limited number of retrospective studies, including a single preprint providing the principal quantitative performance estimates. Therefore, the available evidence currently supports the role of ML primarily as a triage and referral-support approach rather than as a clinically validated replacement for histopathological diagnosis. Future research should prioritise prospective multicentre studies, patient-level external validation, standardised reporting of sensitivity, specificity, and confidence intervals, and inclusion of diverse skin-tone populations to establish the reliability, equity, and real-world applicability of ML-assisted Kaposi sarcoma detection.
