# Supplementary material for: Diagnostic accuracy of machine-learning–based image analysis for early detection of Kaposi sarcoma in people living with HIV
Source: Front Artif Intell. 2026 Sep 14;9:1894174. doi: 10.3389/frai.2026.1894174 (PMC13617372; doi:10.3389/frai.2026.1894174)
Supplement: Supplementary file 1 [file Data_Sheet_1.PDF]

Supplementary Table S1: Complete Characteristics and Evidence Classification of Sources Included in the Narrative Synthesis

No.	Study / Source	Year	Study / Source Type	Population / Setting	Main Focus / Contribution	Evidence Classification
1	**Coates et al. [9]**	2025	Diagnostic accuracy study / preprint	People living with HIV; dark-skinned patients in sub-Saharan Africa	AI-based diagnosis of KS using clinical photographs; reported sensitivity of 89% (95% CI: 85–94%) and specificity of 51% (95% CI: 40–61%)	**Direct KS-specific evidence**
2	**Hussain et al. [14]**	2025	Diagnostic pathology study	KS tissue / skin biopsy samples	Automated detection of KSHV-infected cells using ML-based analysis of immunohistochemical images	**Direct KS-specific evidence**
3	**CDC [1]**	2023	Public-health / surveillance reference	United States / HIV surveillance context	Background information on KS incidence and HIV surveillance	**Contextual evidence**
4	**Cantisani et al. [2]**	2025	Narrative imaging review	Patients with KS	Non-invasive imaging approaches including dermoscopy, dynamic OCT and line-field confocal OCT for KS evaluation	**Contextual evidence**
5	**Akanbi et al. [3]**	2022	Epidemiological study	Adults entering HIV care in Nigeria	KS trends and epidemiological burden among people living with HIV	**Contextual evidence**
6	**Amerson et al. [4]**	2016	Diagnostic accuracy / clinical study	Patients with suspected KS in East Africa	Accuracy of clinical suspicion compared with pathological diagnosis of KS	**Contextual evidence**
7	**Gasmi et al. [5]**	2024	Systematic review	Skin-cancer research populations	ML and deep-learning applications in skin-cancer research; provides broader	**Contextual evidence**

					methodological context	
**8**	**Wei et al. [6]**	2024	Narrative / clinical review	Dermatology / skin-cancer populations	Overview of artificial intelligence applications in skin cancer and implications for diagnostic imaging	**Contextual evidence**
**9**	**Codella et al. [7]**	2017	Image-classification study	Skin-cancer / dermoscopic image datasets	Deep-learning ensembles for melanoma recognition using dermoscopic images	**Contextual evidence**
**10**	**Roche et al. [10]**	2024	Computer-vision evaluation study	HIV self-testing images	Computer-vision AI performance in interpreting HIV self-test images; methodological relevance to AI-assisted HIV care	**Contextual evidence**
**11**	**Aquil et al. [11]**	2025	ML/DL diagnostic study	Diverse skin-tone populations	ML and deep-learning approaches for early detection of skin diseases across diverse skin tones	**Contextual evidence**
**12**	**Kallipolitis et al. [13]**	2025	Systematic literature review	Patients with dermatological conditions	AI-based skin-image analysis for dermatitis, vitiligo and alopecia areata; informs dermatological image-analysis methodology	**Contextual evidence**
**13**	**Muthulakshmi et al. [15]**	2023	Image-classification study	Patients with vascular skin lesions	CNN-based dermoscopic analysis of vascular skin lesions; provides methodological relevance for KS lesion classification	**Contextual evidence**
**14**	**Cantisani et al. [16]**	2023	Imaging evaluation study	Patients with cutaneous KS	Optical coherence tomography for	**Contextual evidence**

					evaluation of cutaneous KS	
**15**	**O'Mahony et al. [17]**	2008	Imaging review / feasibility study	Patients with KS	Imaging techniques for KS, including non-invasive approaches relevant to lesion assessment	**Contextual evidence**
**16**	**Mamimandjiami et al. [18]**	2025	Narrative review	Sub-Saharan African populations	Epidemiology, burden and diagnostic challenges of KS in sub-Saharan Africa	**Contextual evidence**
**17**	**Restrepo et al. [19]**	2006	Radiological review	Patients with KS	Multimodal imaging manifestations of KS and imaging phenotypes relevant to diagnostic interpretation	**Contextual evidence**
**18**	**WHO [20]**	2025	Clinical guideline / reference source	Global HIV care settings	Diagnostic and clinical management context for KS in advanced HIV disease	**Contextual evidence**
**19**	**Salinas et al. [21]**	2024	Systematic review and meta-analysis	Skin-cancer diagnostic populations	AI versus clinicians for skin-cancer diagnosis; contextual evidence for comparative AI diagnostic performance	**Contextual evidence**
**20**	**Sah et al. [22]**	2025	Narrative review	People living with HIV	AI and personalised medicine applications in HIV management and clinical care	**Contextual evidence**
**21**	**Lai et al. [23]**	2025	Systematic review and meta-analysis	Skin-lesion imaging populations	Equity and generalisability of AI for skin-lesion diagnosis using clinical, dermoscopic and smartphone images	**Contextual evidence**

**22**	**Codella et al. [24]**	2019	Benchmark / challenge study	General skin-lesion imaging populations	ISIC skin-lesion analysis and melanoma detection; provides benchmark evidence for dermoscopic ML	**Contextual evidence**
**23**	**Foltz et al. [25]**	2024	Systematic review	Non-melanoma skin-cancer populations	AI applied to non-invasive imaging modalities for skin-cancer identification	**Contextual evidence**
**24**	**Openshaw et al. [26]**	2023	Clinical / disease-classification review	Patients with KS	Taxonomic and immunopathological classification of KS; informs clinical framing and disease interpretation	**Contextual evidence**
**25**	**Udeh & Khatri [27]**	2017	Narrative clinical review	Patients with KS	Clinical indices and diagnostic pathways for KS; supports the need for improved early detection	**Contextual evidence**
**26**	**Jairath et al. [28]**	2024	Systematic review	Dermatological oncology populations	Applications of AI in melanoma and keratinocyte carcinoma diagnosis; broader methodological context	**Contextual evidence**
**27**	**Groh et al. [29]**	2024	Deep-learning diagnostic study	Diverse skin-tone populations	Deep-learning decision support for skin disease diagnosis across skin tones; informs generalisability and equity considerations	**Contextual evidence**

Note: Direct KS-specific evidence refers to primary empirical studies evaluating machine-learning or artificial-intelligence–based image analysis specifically for Kaposi sarcoma. Contextual evidence refers to studies and scholarly sources addressing dermatological AI, skin-lesion image analysis, KS imaging, diagnostic pathways, validation, algorithmic bias, clinical implementation, or related methodological considerations. Contextual sources were used to inform interpretation of imaging, methodological and implementation issues and were not treated as independent demonstrations of KS-specific diagnostic accuracy. Diagnostic performance estimates presented in the manuscript were derived from KS-specific evidence only.
